# Pediatric *tuina* for the treatment of attention deficit hyperactivity disorder (ADHD) symptoms in preschool children: study protocol for a pilot randomized controlled trial

**DOI:** 10.1186/s40814-020-00704-z

**Published:** 2020-11-05

**Authors:** Shu-Cheng Chen, Juan Yu, Lorna Kwai-Ping Suen, Yan Sun, Ya-Zheng Pang, Dong-Dong Wang, Wen-Xia Zhao, Wing-Fai Yeung

**Affiliations:** 1 School of Nursing, The Hong Kong Polytechnic University, 11 Yuk Choi Road, Hung Hom, Kowloon, Hong Kong SAR, China; 2 Pediatric Tuina Health Care Clinic, Shandong University of Traditional Chinese Medicine Affiliated Hospital, Jinan, China; 3 School of Acupuncture, Moxibustion and Tuina, Shandong University of Traditional Chinese Medicine, Jinan, China; 4 College of Traditional Chinese Medicine, Shandong University of Traditional Chinese Medicine, Jinan, China; 5 Beijing Luhe Hospital, Capital Medical University, Beijing, China

**Keywords:** ADHD, Hyperactivity, Anxiety, Sleep disturbance, Pediatric *tuina*, Parent-administered, TCM, Pilot RCT, Protocol

## Abstract

**Background:**

Medication and behavior therapy are the conventional treatments for attention deficit hyperactivity disorder (ADHD), but they have limitations for preschool children. Evidence suggests that pediatric *tuina*, which is a modality of traditional Chinese medicine, might have beneficial effects on this condition.

**Objective:**

To assess the feasibility of conducting an RCT in terms of recruitment, use, and acceptability of the parent-administered pediatric *tuina* for ADHD symptoms in preschoolers.

**Methods:**

It is a single-center, two-arm, parallel, open-label, pilot randomized controlled trial (RCT). Sixty children with pre-specified ADHD symptoms (hyperactivity, anxiety, and sleep disturbance) together with one of their parents will be recruited and randomized into two groups at a 1:1 ratio. Parents in the parent-administered *tuina* group (intervention group, *n* = 30) will attend an online training program to learn pediatric *tuina* skills for ADHD symptoms and conduct this treatment on their children at home. Parents in the parent-child interaction group (comparison group, *n* = 30) will attend an online training about progressive muscle relaxation exercise and do this exercise with their children at home. Additional teaching materials will be provided to the participants in both groups. Both interventions should be carried out every other day during a 2-month treatment period, with each time around 20 min. Assessment will be performed at baseline, week 4, and week 8. The primary outcome measure is the Swanson, Nolan, and Pelham parent scale; the secondary outcomes include preschool anxiety scale, children’s sleep habits questionnaire, and parental stress scale. A process evaluation embedded within the outcome evaluation will be performed. Differences in the scale scores and test parameters between groups will be examined using a linear mixed-effects model. Qualitative data will be analyzed using thematic content analysis, facilitated by QSR NVivo.

**Discussion:**

This study will provide evidence on the acceptability and feasibility of pediatric *tuina* for ADHD in preschool children. The process evaluation will help to better understand the facilitators and barriers of the intervention functioning.

**Trial registration:**

The study was registered at ClinicalTrials.gov (Identifier: NCT04237259) on 14 February 2020.

Protocol version: 2; date, 23 June 2020

## Background

Attention deficit hyperactivity disorder (ADHD) is a prevalent neurobehavioral disorder of childhood period, which is characterized by chronic symptoms of children’s developmentally age-inappropriate, undesirable features and can be accompanied by academic and behavioral problems [1]. The overall incidence rate in children aged from 4 to 17 years in the USA was approximately 10.2% in 2015-2016, and the prevalence of diagnosed ADHD children in the USA has a continued significant increase in the past 20 years [2]. Children diagnosed with ADHD usually suffer from negative social and academic problems [3, 4], and more than half of them have ADHD comorbidities (e.g., anxiety, depression) [5, 6]. Moreover, as a chronic medical condition, ADHD symptoms may persist into adulthood [7–9].

Medication therapy and behavior therapy are recommended by the American Academy of Pediatrics (AAP) for ADHD [10]. Medication has a beneficial and rapid effect on ADHD core symptoms, but the effect does not maintain if the treatment discontinues. Besides, medication treatment, both stimulants, and non-stimulants may lead to potential adverse side effects [10–12]. Medication has a beneficial and rapid effect on ADHD core symptoms, but the effect does not maintain if the treatment discontinues. Besides, medication treatment, both stimulants, and non-stimulants may lead to potential adverse side effects [10]. Evidence-based behavioral treatments are strongly recommended for ADHD children of all ages. It provides parents with more chances to interact with their children. However, some factors limit the popularization of this therapy, such as the high cost, high family involvement, parents’ learning abilities, and the lack of standardized training patterns [10, 13]. Complementary therapies are also commonly used by children and adolescents with ADHD symptoms [14], and pediatric *tuina* is one of the complementary therapies.

**Fig. 1:**
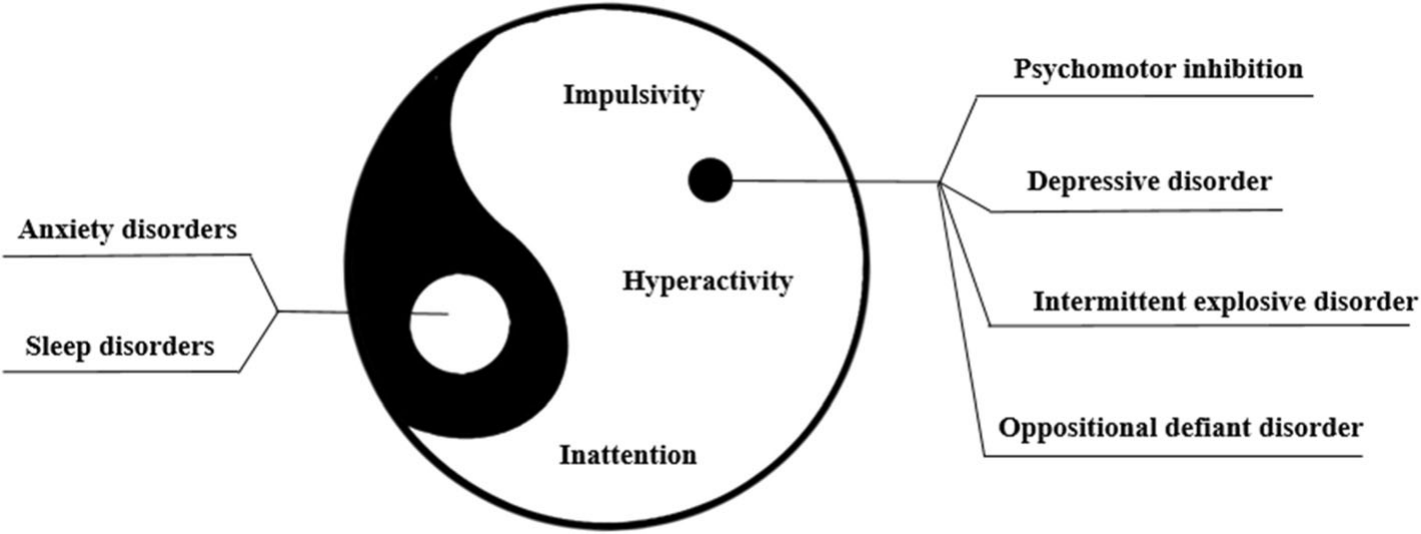
Illustration of the pathological mechanism of ADHD based on TCM theory

Pediatric *tuina*, which is also called traditional Chinese medicine (TCM) pediatric massage or pediatric *anmo*, is a special modality of TCM therapies. It is a manual intervention for preventing and treating medical conditions in children based on TCM theories and previous experience. Different from other massage types, the body part to be treated by pediatric *tuina* could be a point, a line, a circle, or an area; thus, the corresponding manipulations are more complex. Commonly used *tuina* techniques include pushing, circular rubbing, nipping, kneading, pushing, arc-pushing, twisting, and rotating. Pediatric *tuina* is usually used for asthma, respiratory infection, congenital muscular torticollis (CMT), diarrhea, constipation, anorexia, atopic eczema, and other medical conditions in infants and children [15–21]. In the perspective of TCM, ADHD symptoms are associated with the disharmonious situation between *yin* and *yang* of a body. An ancient TCM literature—“Ling Shu·The Application of the Needles”—recorded relevant symptoms as follows: “Double *yang* persons, their spirit is easily excited, and their *qi* proceed easily, they are fervid and lofty, speak well and fast. When walking they raise their feet high.” Most researchers believe that the pathogenesis of ADHD is the abnormal exuberance of *yang*, which can be relieved by specific approaches of massage [22, 23], and the corresponding principle is to adjust the excess, deficiency, and inter-impairment of *yin* and *yang* to return to the balanced state. *Yin* and *yang* are mutual rooting and may induct, contain, transform to each other, which explains why *yang* syndrome could comprise some comorbidities like anxiety disorders, oppositional defiant disorder, psychomotor inhibition, and intermittent explosive disorder (Fig. 1).

Parent-administered interventions attempt to enhance a parent’s understanding of child behavior management and the quality of the parent-child interactions, with the ultimate goal of optimizing the child’s developmental course [24]. A systematic review and meta-analysis of non-pharmacological interventions for ADHD found that psychological treatments administered by parents showed the largest effects among 54 randomized controlled trials (RCTs) for preschool children in terms of ADHD symptoms improvements [25]. A recent RCT of a dual-parent and trainer-delivered *qigong* massage intervention for young children with autism indicated that the parent-delivered massage provided effective early intervention for autism that was feasible for delivery at home [26]. Hence, the combination of parent-administered intervention mode and pediatric *tuina* might be a cost-effective and feasible way of treating ADHD symptoms.

Studies have been done to examine the effects and safety of complementary therapies on ADHD. Catalá-López and colleagues conducted a systematic review to compare the efficacy and safety of pharmacological, psychological, and complementary and alternative therapies for ADHD stressed that high-quality studies on multiple treatments for ADHD in children and adolescents are urgently needed [27]. Chen and colleagues carried out an RCT on using TCM acupressure therapy for treating ADHD in children, leading to a conclusion that TCM acupressure therapy had better effects than *Ritalin* on ADHD symptoms with higher acceptability [28]. Recently, Chen and colleagues performed a systematic review of using massage therapy for ADHD in children and adolescents, suggesting that massage seemingly has beneficial effects for improving ADHD symptoms However, the methodological quality of the included clinical trials was generally poor. Limitations include unclear randomization and allocation concealment methods, incomplete reporting of outcome data, a lack of validated outcome measures, and a high risk of biases correlated to a failure to blind participants and outcome assessors [29]. Further rigorously designed clinical trials are warranted to assess the effects of pediatric *tuina* on ADHD. Furthermore, there is no RCT conducted to test the feasibility and effects of parent-administered pediatric *tuina for* ADHD. Our team has demonstrated that it is feasible to train patients to perform self-administered acupressure for insomnia [30], knee pain [31], and stress [32] by a short training program. This pilot study will test the feasibility of training parents to conduct *tuina* on their children. Therefore, we designed this pilot study to explore the feasibility of parent-administered pediatric *tuina* for ADHD in preschoolers.

## Objectives

The primary aim of the pilot trial is to assess the feasibility of parent-administered pediatric *tuina* for ADHD symptoms (hyperactivity, anxiety, and sleep disturbance) in preschool children for a further fully powered RCT. Specifically, the objectives are the following:

1. To test the feasibility of the study design (such as participant recruitment, participants’ compliance, and data collection procedures).
2. To access the use and acceptability of parent-administered pediatric *tuina* intervention.
3. To evaluate the adverse events of the parent-administered pediatric massage for both preschool children with ADHD symptoms and their parents.
4. To compare the preliminary effects of parent-administered pediatric *tuina* with parent-child interaction training control intervention.

## Methods

The present protocol has been registered within ClinicalTrials.gov (Identifier: NCT04237259) and is being reported in accordance with the reporting guidance provided in the Standard Protocol Items: Recommendations for Interventional Trials (SPIRIT) statement [33] (see checklist in Additional file 1). The final study report will be prepared in accordance with the reporting guidance provided in the CONSORT extension for reporting pilot randomized controlled trials [34, 35].

### Trial design

This study is a single-center, two-arm, parallel, open-label, pilot randomized controlled trial. Participants (parent-child pairs) will be distributed into the intervention group and the control group at a 1:1 ratio. Parents in the intervention group (*n* = 30) will receive a training of parent-administered pediatric *tuina* and perform manipulations on their child. Parents in the active control group (*n* = 30) will be given an online training session about the skills of interacting with their children and a progressive muscle relaxation exercise. Treatments as usual (TAU) of the preschoolers in both groups will not be intervened during the treatment period.

This study includes a feasibility phase, which is expected to be the first 2 months of data collection treatment period with around eight parent-child pairs. The feasibility stage is designed to preliminary test the logistics of the intervention delivery, and to undertake an early evaluation of the study design of the whole pilot trial.

### Participant timelines

**Fig. 2:**
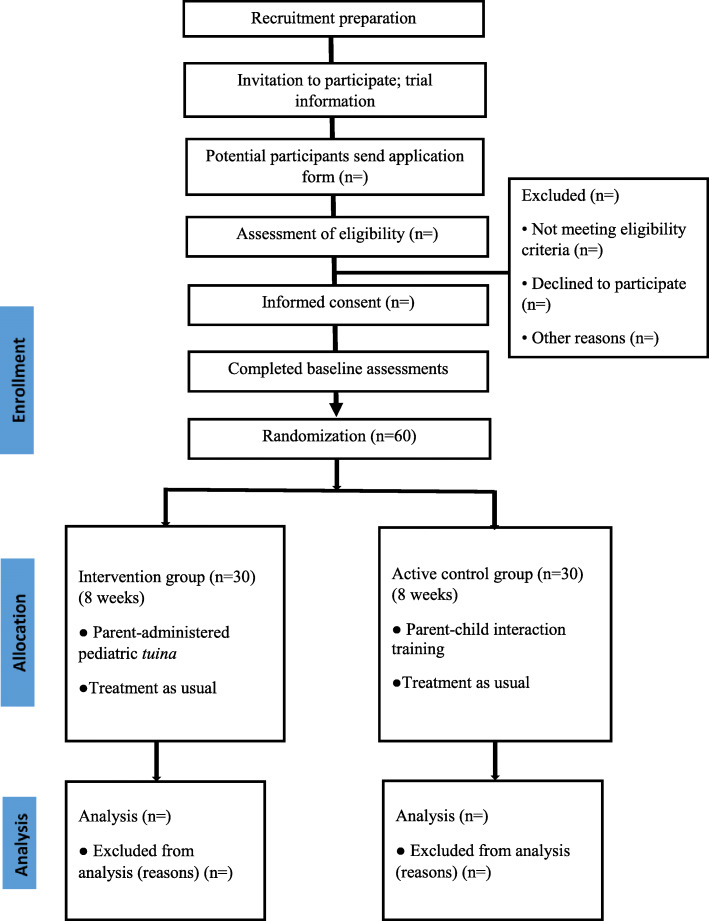
CONSORT flow diagram

The treatment period will be 8 weeks. Outcomes will be measured at 3 time points: baseline, week 4, and week 8. A CONsolidated Standards of Reporting Trials (CONSORT) flow diagram demonstrates the procedure of the trial, including the recruitment, allocation, and intervention (Fig. 2). A Standard Protocol Items: Recommendations for Interventional Trials (SPIRIT) diagram shows the schedule of enrollment, intervention, and assessment (Table 1).

**Table 1:**
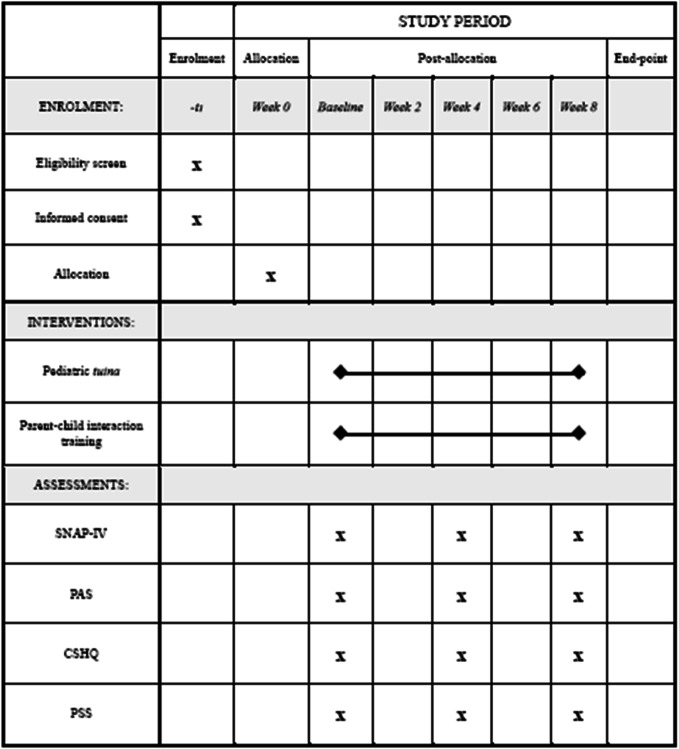
SPIRIT diagram

### Study setting

Participants will be recruited at Shandong University of Traditional Chinese Medicine Affiliated Hospital, which is a tertiary A hospital with a pediatric *tuina* clinic center and a pediatric *tuina* teaching base. The hospital is located in Jinan City, Shandong Province, China.

### Subjects and recruitment methods

This study will recruit 60 preschool children with ADHD symptoms (hyperactivity, anxiety, and sleep disorders) together with their parents. The methods of recruitment are as follows:

1. Flyers and posters in the affiliated hospitals of the university
2. Advertisement through mass media, including newspaper and radio
3. Poster through social media, including WeChat and Weibo

Children and their parents who are interested in participating will be screened according to the eligibility criteria via an online meeting. The researcher who is responsible for recruitment will explain the procedure of the study to the subjects’ parents. Parents will be required to sign the informed consent before participating in this study if they are eligible.

### Sample size

There are few published guidance concerning how large the sample size a pilot study should be. “Rule of 12” for continuous variables recommends that at least 12 participants for pilot studies with the primary focus of estimating average values and variability for planning larger subsequent studies. This size is practical for most early-stage researchers within a single center while still providing valued preliminary information [36]. It has been proposed that a sample size ranged from 10 to 40 each group is sufficient to assess the adequacy in providing a precise estimate of the between-group effect size. Assuming a 25% dropout rate, the total sample size will be 60 (*n* = 30 in the intervention group, *n* = 30 in the active control group). A sample size of at least 30 participants in each group will help to yield a confidence interval whose lower limits can help define the range of plausible values for subsequent power analysis [37]. Such a sample size is considered to be sufficient to provide the preliminary data required to test the feasibility, acceptability, and effects of parent-administered pediatric *tuina* for preschool children and assess the study design to provide instructive information and guidance to the further fully power study.

### Eligibility criteria

#### Inclusion criteria

Inclusion criteria for the children are the following:

1. Children between 3-7 years old by the start of the assessment
2. Having a score equal to or higher than the borderline cutoff of the Swanson, Nolan, and Pelham Parent Rating Scale (SNAP) [38], indicating the children had moderate ADHD symptoms

Children with ADHD symptoms of hyperactivity, anxiety, or sleep disturbance will be recruited in this study. We will not restrict the children to have a formal medical certificate since children are usually assessed and diagnosed by professionals after 7 years old; hence, their hyperactivity, anxiety, and sleep disturbance symptoms have not yet received a diagnosis. We will consider these children according to the inclusion criteria.

Inclusion criteria for parents are the following:

1. Able to communicate using Mandarin
2. Willing to learn the knowledge and manipulations of pediatric *tuina* for ADHD symptoms
3. Available to take their children to the designated hospital for pattern identification and conduct manipulations at home according to the study process
4. Agree to give informed consent

#### Exclusion criteria

Exclusion criteria for children are the following:

1. Currently receiving other massage therapies
2. Having other developmental disability (e.g., autism spectrum disorders, intellectual disability)
3. Having acute infection diseases (e.g., scarlet fever, chickenpox), hemorrhagic diseases (e.g., bleeding, local places of various kinds of malignant tumor), or dermatological problems (e.g., scald, severe skin lesion, skin infections)
4. Having any severe illness or medical condition that the investigator deems not appropriate to receive pediatric *tuina* (e.g., bone fractures, paraplegia)

Exclusion criteria for parents are the following:

1. Having any severe psychiatric disorder (e.g., major depressive disorder)
2. Having difficulties to conduct massage therapy due to physical problems
3. Unable to imitate the manipulations in the videos provided to test the cognitive and learning abilities

#### Withdrawal criteria

Withdrawal criteria for children are the following:

1. Occurring of severe adverse events
2. Commencement of other pharmacological or psychological therapies for ADHD symptoms during the treatment period
3. Receiving a major surgical intervention that leads to unsuitability for receiving pediatric *tuina*

Withdrawal criterion for parents is the following:

1. Withdrawal of consent

### Participant recruitment procedure

A total number of 60 children aged 3-7 years will be recruited with one of their parents in this study. Figure 2 shows the recruitment procedure and based on the CONSORT guidelines. A research coordinator will be responsible for obtaining written informed consent and face-to-face screening. Detailed procedures are the following:

#### Recruitment and preliminary screening

The study will be promoted via posters, emails, and phone messages. Contact methods of a research coordinator will be provided. Parents who intend to participate in the study are required to contact the research coordinator via telephone call or WeChat for preliminary screening. Potentially eligible subjects who meet the preliminary criteria will be invited to further procedures.

#### Informed consent and screening

Potentially eligible participants will receive an interview online. A research coordinator will explain the study to the parents and obtain the written informed consent. Further assessments will be conducted based on inclusion eligibility criteria. Parents will be required to imitate two manipulations in a video that is provided by the research coordinator, and they will be excluded if they fail to do imitation. Personal information about participants will be collected. A case report form (CRF) will be performed by the research coordinator.

#### Assigning a study schedule

The research coordinator will assign the baseline assessment, intervention, and follow-up schedule to the eligible participants.

### Sequence generation and allocation

Children with their parents will be randomly divided into the intervention group or the active control group after the completion of baseline assessments. Block randomization will be carried out using MS Excel with a random block size of 4 to 8 at a 1:1 ratio. The allocation code will be enclosed in sequentially numbered opaque envelopes and kept by a coordinator and will dispense a code to each participant upon confirmation of completion of baseline assessment by the research coordinator. The research coordinator will be blind to the study arms, and the PI will not be involved in the post-baseline data collection.

### Blinding

Due to the nature of the intervention (pediatric *tuina*) and the active control intervention (parent-child interaction training), it is impossible to blind the practitioners and the subjects. Therefore, they will not be blinded. However, parents might have questions when filling the rating scales. If they have any problems, they can inquire to the research outcome assessors. For those outcome assessors who are in charge of guiding the parents to fill the questionnaires and collecting data, they will be blinded.

### Interventions

#### Intervention group: parent-administered pediatric tuina

**Fig. 3:**
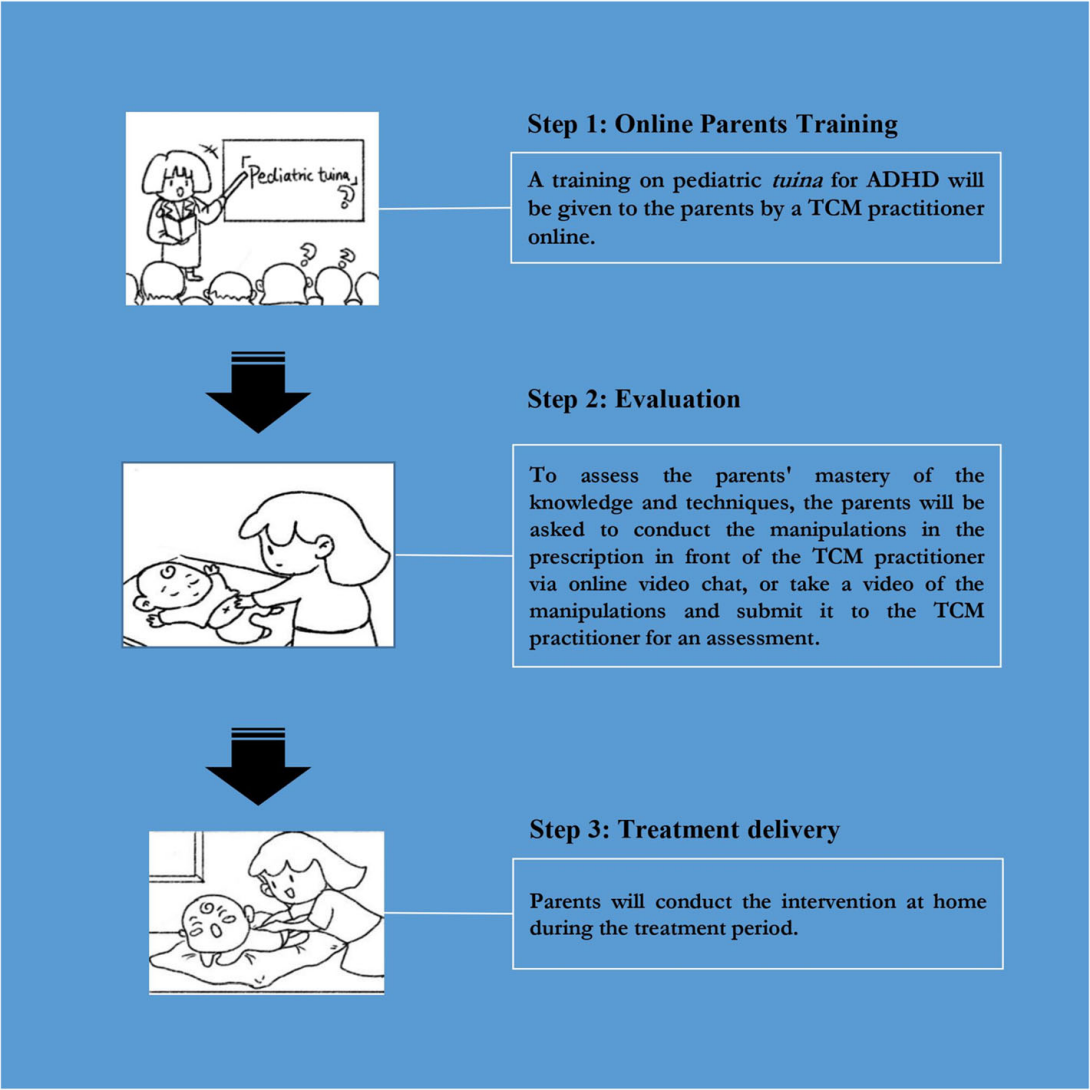
Illustration of intervention procedure to parents

Children in the intervention group will receive parent-administered pediatric tuina during the treatment period. Figure 3 presents the intervention implementation procedure. The parents will attend an online training program (2 training sessions, 5 h in total) to gain the knowledge and basic manipulations of pediatric *tuina* on ADHD symptoms in preschoolers. Contents of the training sessions will cover the manipulations and acupoints that are commonly used in treating ADHD. The instructor will, inspect their practice, and make feedback to them if there is any deviation. At the end of the training sessions, to assess the parents’ mastery of the contents and techniques, the parents will be asked to conduct the manipulations in the prescription (see Additional file 3) in front of the TCM practitioner via online video chat, or take a video of the manipulations in the prescription and submit it to the TCM practitioner for an assessment. Parents who have passed the assessment could the start conducting the intervention at home, while for those who have not passed, they will be asked to receive the training for one more time until they could appropriately conduct the manipulations. Then they will deliver the *tuina* on their child at home in the following 2 months according to the *tuina* prescriptions formulated by the TCM practitioner. The *tuina* prescriptions will comprise the acupoints selected, the corresponding manipulation, conduction frequency, times of each manipulation, and other necessary information. The parents will be told to deliver pediatric *tuina* therapy on their children every other day, with 20-30 min each day. A logbook will be used for recording (see Additional file 4). Videos of the manipulation techniques and relevant teaching materials will be provided to the parents via social media.

#### Active control group: parent-child interaction training

Parents in this group will receive a parent-child interaction training program which includes progressive muscle relaxation exercise training for children and a health education booklet of ADHD and coping skills. The parents in this group will have to complete an online training session of progressive muscle relaxation exercise [39], which aims to help children reach a relaxation state by relaxing their muscles when facing stresses. The health education booklet covers the background knowledge of ADHD, suggestions, and examples of coping skills (e.g., coping skills for reaction to stress and conflict with children) and interaction skill training for parents (e.g., behavior management, self-regulation, improving organization, improving relationship, and use of encouragement) [40]. All the training contents are recommended by the Department of Health, the government of the Hong Kong Special Administrative Region. The training serves as an early intervention, with the emphasis on modifying the improper parenting methods and enhancing the positive parent-child interaction when the children are still at their preschool stage. Parents will be asked to complete an online written test after they have viewed the online training and teaching materials. They will be asked to spend at least 20 min every other day during the treatment period on doing progressive muscle relaxation exercises with their child and record their practice on a logbook (see Additional file 5).

During the study period, the children’s ongoing treatment, such as medications, behavioral therapies, and other services such as mental health service and parents-supporting activities will not be intervened in both groups. Information on their TAU will be recorded, and they will be asked not to start new treatment targeting ADHD symptoms during the study period. For subjects in both groups, they will be considered to have completed the program (completers) if they have attended the training lessons and performed the treatment at home for at least 3 days per week during the 8-week intervention period.

#### Intervention modifications

The manipulations selected are gentle and safe for preschoolers. It is unlikely that pediatric *tuina* leads to severe adverse events. Any pain or discomfort, if any, during the intervention, are transient and will not bring to serious sequela. If participants withdraw during the treatment period, their reasons of withdrawal and adverse events will be recorded. They will be followed-up until the adverse events resolved.

#### Intervention adherence

For parents in both groups, a consultation will be available for clarification or helps during the treatment period through WeChat. We propose to use a combined approach that we will instruct the parents to perform the tuina intervention every other day and at least 3 times per week. Parents are required to record their massage practice on a logbook during the treatment period, and a researcher will remind them of carrying out the interventions two times a week. To encourage the subjects to complete the treatment, we will provide them with the intervention of the other group as a reward if they have completed the allocated treatment. During the whole process of intervention delivery and data collection, relevant information, international news, scientific research achievements, and other useful resources will be shared with the subjects, and those subjects who finish the whole process of the study will receive the resources 2 or 3 times a week until 6 months after the endpoint of the trial. A process evaluation will be carried out to better adjust the intervention acceptability and feasibility [41].

## Outcome measures

### Feasibility outcomes

The study will assess the following feasibility outcomes throughout the 8-week study period: (1) recruitment rate; (2) consent rate; (3) adherence to the intervention; (4) completion of study assessments, and (5) adverse events. Participant satisfaction will be assessed by parent self-report questionnaire and focus group interviews in the process evaluation.

### Patient-centered outcomes

Patient-centered outcomes include the following primary outcomes and secondary outcomes, which will also be used in the future main trial.

#### Primary outcome measure

##### Swanson, Nolan, and Pelham parent rating scale (SNAP-IV-P)

The SNAP-IV Chinese Version ADHD Scale for parents is the primary outcome measure in this study. It has 26 items that include 18 ADHD symptoms (nine inattentive, nine hyperactive/impulsive) specified in the DSM-5. Items are rated for frequency by the parents on a 4-point scale (0 “not at all” to 3 “very much”) and average ratings per item are then calculated for each subscale. The Chinese version shows satisfying test-retest reliability (intraclass correlation = 0.59), internal consistency (*α* = 0.88), concurrent validity (Pearson correlations = 0.56), and discriminant validity [38].

#### Secondary outcome measures

##### Preschool anxiety scale (PAS)

The PAS is a 34-item parent-rated scale, which assesses anxiety symptoms in young children across six subscales: panic attack and agoraphobia, separation anxiety, physical injury fears, social phobia, obsessive-compulsive, generalized anxiety/overanxious disorder. The measure has sufficient evidence supporting construct validity, and the total score and subscales have great internal consistency (*α* = 0.72-0.92) [42]. Wang et al. evaluated the applicability of PAS in China and concluded that PAS had acceptable psychometric properties, and applicable to assess anxiety syndrome of preschoolers in China. Correlations between each item and its subscales ranged from 0.50 to 0.78. Cronbach’s alpha coefficient, the split-half reliability coefficient, and the test-retest coefficient of the scale were 0.87, 0.83, and 0.70 respectively [43].

##### Children’s sleep habits questionnaire (CSHQ)

The CSHQ is a retrospective, 33-item parent questionnaire that has been used in several studies to examine sleep behavior in young children. Previous studies suggested that the CSHQ’s structure needs to be adjusted to accommodate the preschool children in China, and should be used with great caution [44, 45]. A final 4-factor structure CSHQ was suggested that compromised 28 items, with bedtime behaviors (*α* = 0.59), sleep behaviors (*α* = 0.62), morning wakings (*α* = 0.69), and daytime sleepiness (*α* = 0.67). The four factors represented improvements to the psychometric quality of the CSHQ and the reliability of subscales was all acceptable. Therefore, according to the studies above, we will use the revised 28-item CSHQ for measuring the sleep quality of the children [45].

##### Parental stress scale (PSS)

The PSS is a measure of “individual differences in the level of stress associated with raising children,” which includes 18 items on the perception of parental stress and uses a Likert style rating scale [46]. The factor structure of the PSS was validated and manifested satisfactory internal consistency (*a* = 0.84). There was evidence of convergent validity with family functioning (*r* = −0.51), parental anxiety (*r* = 0.44), and depression (*r* = 0.35). Discriminant analyses showed low, non-statistical correlations with domains of children’s life quality (*r* = −0.07-−0.18) [47]. The Chinese version was validated by 257 Hong Kong Chinese parents with at least one child under 12 years, and the reliability was 0.89 [48].

## Process evaluation

**Fig. 4:**
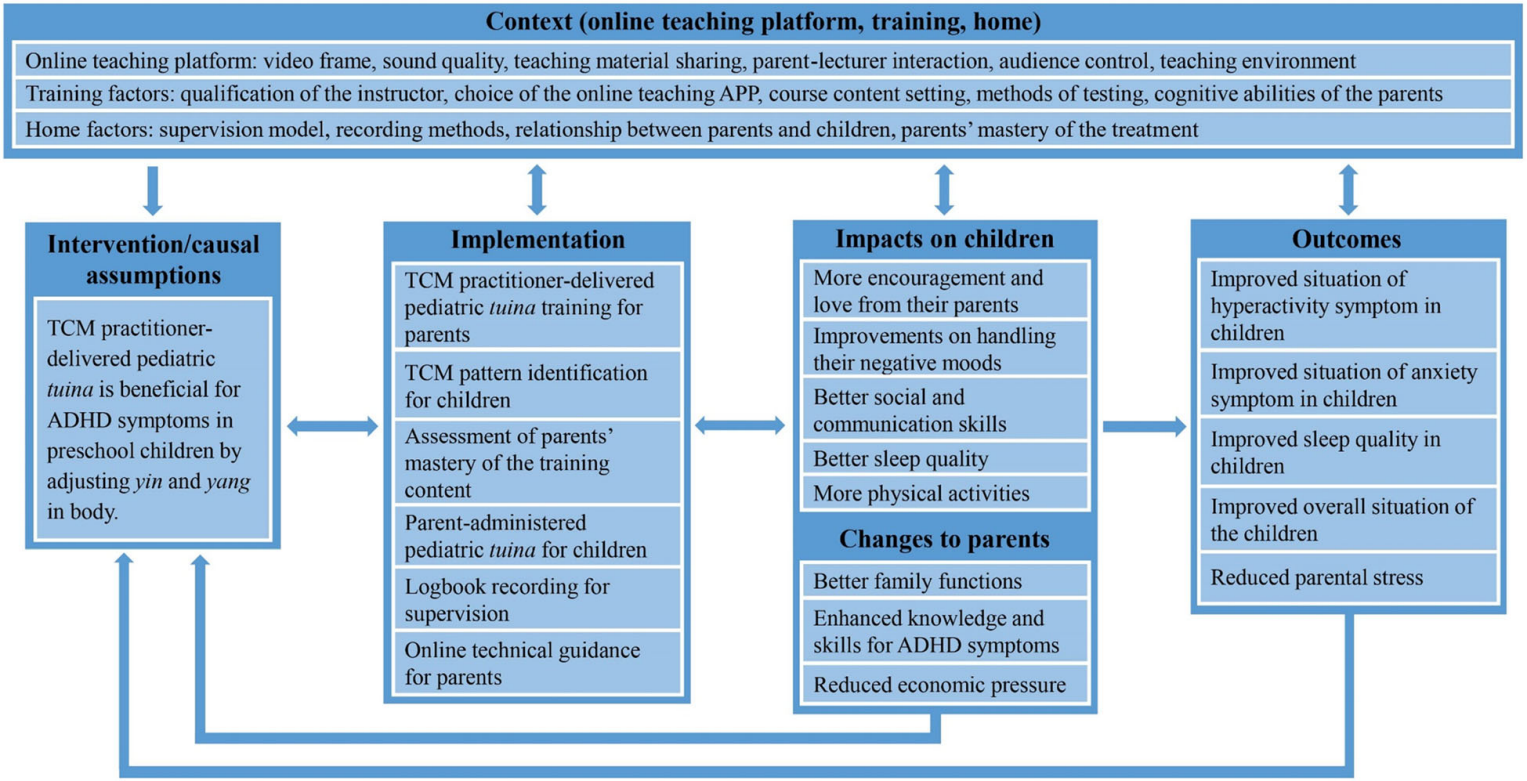
The parent-administered pediatric tuina logic model

Process evaluation is critical for interpreting and understanding the functioning of the intervention, which includes implementation, mechanisms, and context [49]. The parent-administered pediatric *tuina* therapy is a complex intervention. Parents will be trained in the hospital and perform the treatment on their children at home, and the *tuina-*prescriptions are individualized. These components highlight the requirements for investigating how implementation can be achieved across various contexts. Figure 4 shows a logic model we use to visually present the content of the intervention and its causal assumptions, the implementation, the contexts in which it is delivered, the potential impacts considered, as well as the outcomes it aims to achieve. A qualitative process evaluation embedded within the outcome evaluation will be performed to better understand the facilitators and barriers of the intervention functioning (Table 2).

**Table 2:** Overall design of process evaluation

	Qualitative data		
	Self-report questionnaire	Focus group interview	Parents logbook
**Parents**	✓	✓	✓
**Practitioners**	✓		
**Research assistants**	✓		
**Outcome assessors**	✓		

### Data collection

Qualitative data will be collected through self-report questionnaires, focus group interviews, and parents’ written logs. A researcher will be in charge of the interviews. Parents will be informed of the qualitative process evaluation at the time of enrollment. Reflection from each interview and self-report will facilitate the subsequent data collection process.

#### Self-report questionnaire

The self-report questionnaires will gather information about the intervention delivery experience, facilitators, barriers, expectations, recommendations, and sustainability of the intervention. Parents will fill out the questionnaires in week 4 and week 8. TCM practitioners, research assistants, and outcome assessors will complete this assessment in week 8. Emphasis will be placed on the aspects of intervention delivery, feasibility, and acceptability.

#### Focus group interviews

Semi-structured interactive focus group interviews among parents will be conducted after the feasibility stage, providing them with the chance to articulate intervention delivery experience. This method will investigate parents’ perceptions of the intervention, and their opinions on what works or does not work about commencing the intervention. Questions will relate to the barriers and facilitators toward implementation, and what would be expected in the future to sustain and promote the intervention.

#### Parent logbooks

Parents’ logbooks will be used for supervising the performance of pediatric *tuina* delivery. Parents will be responsible for completing weekly logs online, specifying the acupoints locations, manipulations, duration, frequency, and their queries. An outcome assessor will review the logs and send reminders for the completion of logs where necessary.

## Data analysis and management

### Statistical analysis

Descriptive statistics for sociodemographic variables and clinical variables will be presented. Data will be presented as mean and standard deviation. The feasibility outcomes will be presented as percentage. The SNAP scores between the intervention group and the active control group in week 4 and week 8 will be compared using a linear mixed-effects model. Other analyses include using a linear mixed-effects model to compare the SNAP (for measuring ADHD symptoms), PAS (for measuring anxiety), CSHQ (for measuring sleep disturbance), and PSS (for measuring parental stress) in the intervention group with the control group at all assessment time points. To estimate the changes with time in each group, the repeated measures ANOVA will be used. The effect sizes will be estimated using between-group mean difference divided by a pooled standard deviation.

The analyses will be based on the intention-to-treat approach. Completer analysis will be a secondary analysis. Completer is defined as those who have attended all lessons, practiced the pediatric *tuina* or parent-child interaction training at least 3 times per week during the treatment period, and completed assessment time point. As the sample size is underpowered, all analysis will be presented with 95% confidence intervals to inform the precision of the results. Since data at the pilot stage is preliminary, it should be treated with caution.

All qualitative data, including interview transcriptions, text from self-report questionnaires, and content of parent logbooks, will be analyzed using thematic content analysis, facilitated by NVivo (QSR, Melbourne, VIC). Adaptations and rationale behind all reported changes to the intervention will also be listed, analyzed, and summarized. All interviews will be audio-recorded and transcribed verbatim. The transcripts will be verified for accuracy against the sound files and revisions will be made as appropriate. Any identifiable remarks will be anonymized before transcripts being imported into NVivo.

### Date management

Data will be collected through e-questionnaires, which will be kept strictly confidential, and could only be used for purposes related to this study. Data collected will be stored in our data system for 3 years and destroyed after that. Participants have the right to use their personal data, such as inspection, preservation, management, disclosure/non-disclosure, erasure, and/or in any way dealing with or disposing of any of their personal data in or for this study.

## Discussion

This pilot trial aims to assess the feasibility of parent-administered pediatric *tuina* for ADHD symptoms in preschool children. There have been several studies conducted on pediatric *tuina* for ADHD; however, those studies were limited by various methodological problems such as invalidated outcomes measures, unclear randomization and allocation concealment methods, lack of appropriate control intervention to test the specific effects. To more rigorously assess the preliminary effects of pediatric *tuina*, we designed this pilot trial to overcome the limitations of previous studies.

Children in the intervention group will receive individualized *tuina* prescriptions. It might increase the complexity of the intervention and hamper the reproducibility of the intervention, but treatment individualized based on TCM pattern identification is believed to be the optimal practice of pediatric *tuina*. Our design will simulate the real-world practice of *tuina* in the clinical setting.

The pediatric *tuina* is a complex intervention based on TCM theory and past clinical experience, of which the theoretical framework is relatively less researched. Therefore, we included a process evaluation and proposed a logic model to depict the possible causal assumption and mechanism of impacts of pediatric *tuina*. This logic model will inform further fully-powered study and guide trials with similar parent-administered settings in future research.

We use parent-child interaction training as a control intervention which includes the progressive muscle relaxation exercise and health educational materials. The use of sham acupressure as a control intervention is difficult as the non-acupoint locations are not well defined [50]. This control design offers training on muscle relaxation and coping skills, and education of ADHD knowledge to the parents. Both groups contain components that aim to improve parent-child interaction, relive children’s ADHD symptoms, and enhance parents’ knowledge of ADHD. This controls the non-specific effects of pediatric *tuina* due to enhanced parent-child interactions and contacts time to the research personnel.

We anticipate that there will be a few practical issues in performing this study. Firstly, the subjects will be provided with two consultations for TCM pattern identification (one at baseline and one at the end of first month) and the *tuina* prescription may be adjusted. However, the subjects may be more preferable to the original manipulation that they learned at the beginning as they have already mastered the manipulation and found benefit effects. In this case, we may offer them an option to continue the original manipulation. Secondly, parents usually have a great commitment to their family and their time schedule may be less flexible. Although we will inform them the schedule of the training lessons before baseline assessment, we foresee that some parents may not be able to attend all the lessons after randomization.

There are several limitations to this pilot study. First, given the nature of the intervention and the control intervention, it is impossible to blind either the parents, children, or TCM practitioners. To minimize the detection bias, the TCM practitioners would not be involved in the outcome assessment and the outcome assessors will be blinded during the process of intervention conduction and data collection. Second, although we will use MoCA to ensure that the parents have a normal cognitive function, the discrepancies in the learning abilities and education level may affect their mastery of the *tuina* technique and hence the effects of the treatment. However, it would truly reflect the implementation of the intervention in the daily clinical practice as the parents’ backgrounds usually vary. Finally, our control group did not test the *tuina’s* specific effect of exerting physical pressure on the specific body location based on meridian theory.

Our study will add to the current evidence of pediatric *tuina* for ADHD in preschool children based on meridian theory. Parent-administered pediatric *tuina* is a potential alternative therapy with fewer side effects and economic burden, and is more flexible for parents and children to receive the treatment. Our results will also inform the clinicians and practitioners about the feasibly of training the parents to administer the *tuina* technique to relieve their child’s ADHD symptoms.

## Access to data

Researchers from Hong Kong Polytechnic University and Shandong University of Traditional Chinese Medicine Affiliated Hospital involved in this trial will have access to the final trial dataset.

## Dissemination

Dissemination of the results of the study will be carried out via submitting to the peer-reviewed journals, presenting on academic workshops and conferences, providing online or face-to-face courses for parents, and demonstrating the results on social media sites.

## Study status

The trial protocol was approved on 18 September 2019 and registered in ClinicalTrial.gov (NCT04237259) on 14 February 2020 (Additional file 2). The trial is currently open for recruitment. The study will be tracked by the ethics committee of Shandong University of Traditional Chinese Medicine Affiliated Hospital and the Departmental Research Committee of the Hong Kong Polytechnic University, and will be audited every 12 months.

## Supplementary Information

**Additional file 1:** SPIRIT Checklist.

**Additional file 2:** World Health Organization Trial Registration Data Set.

**Additional file 3: **Sample of a pediatric *tuina* prescription.

**Additional file 4: **Parent logbook of pediatric *tuina* group.

**Additional file 5:** Parent logbook of parent-children interaction group.

## Data Availability

Not applicable.

## Abbreviations

ADHD: Attention deficit hyperactivity disorder
AAP: American Academy of Pediatrics
TCM: Traditional Chinese medicine
CMT: Congenital muscular torticollis
RCT: Randomized controlled trials
SNAP: Swanson, Nolan, and Pelham
CONSORT: Consolidated Standards of Reporting Trials
TAU: Treatment as usual
SPIRIT: Standard Protocol Items: Recommendations for Interventional Trials
CRF: Case report form
CIFG: Comprehensive Intervention Fidelity Guide
PAS: Preschool anxiety scale
CSHQ: Children’s sleep habits questionnaire
PSS: Parental stress scale
