# Supplementary material for: Pediatric tuina for the treatment of attention deficit hyperactivity disorder (ADHD) symptoms in preschool children: study protocol for a pilot randomized controlled trial
Source: Pilot Feasibility Stud. 2020 Nov 5;6:169. doi: 10.1186/s40814-020-00704-z (PMC7643336; doi:10.1186/s40814-020-00704-z)
Supplement: Supplementary file 2 — Additional file 2: World Health Organization Trial Registration Data Set. [file 40814_2020_704_MOESM2_ESM.docx]

**World Health Organization Trial Registration Data Set**

| Data category | Information |
| --- | --- |
| Primary registry and trial identifying number | ClinicalTrials.gov NCT04237259 |
| Date of registration in primary registry | 14 Feb, 2020 |
| Secondary identifying numbers | ChiCTR2000032495 |
| Source(s) of monetary or material support | No funding |
| Primary sponsor | N/A |
| Secondary sponsor(s) | N/A |
| Contact for public queries | CHEN Shu-Cheng, MA [852-67653381] [shucheng.chen@connect.polyu.hk] |
| Contact for scientific queries | YEUNG Wing-Fai, PhD [852-27664151] [jerry-wf.yeung@polyu.edu.hk]  The Hong Kong Polytechnic University; 11 Yuk Choi Road, Hung Hom, Kowloon, Hong Kong SAR, China |
| Public title | Pediatric *Tuina* for the Treatment of Attention Deficit Hyperactivity Disorder (ADHD) Symptoms in Preschool Children |
| Scientific title | Pediatric *Tuina* for the Treatment of Attention Deficit Hyperactivity Disorder (ADHD) Symptoms in Preschool Children: a Pilot Randomized Controlled Trial |
| Countries of recruitment | China |
| Health condition(s) or problem(s) studied | Attention deficit hyperactivity disorder symptoms |
| Intervention(s) | Experimental intervention: Parent-administered pediatric *tuina* (20-30 minutes per time)  Active comparator: Parent-child interaction training (20-30 minutes per time) |
| Key inclusion and exclusion criteria | Ages eligible for study: 3-7 years |
|  | Sexes eligible for study: both |
|  | Accepts healthy volunteers: no |
|  | Inclusion criteria for the children are: children between 3-7 years old by the start of the assessment; having a score equal to or higher than the borderline cutoff of the Strengths and Weaknesses of ADHD Symptoms and Normal Behaviors Rating Scale (SWAN), indicating the children had moderate ADHD symptoms. Inclusion criteria for parents are: able to communicate using Mandarin; willing to learn the knowledge and manipulations of pediatric *tuina* for ADHD symptoms; available to take their children to the designated hospital for pattern identification and conduct manipulations at home according to the study process; agree to give informed consent. |
|  | Exclusion criteria for children are: currently receiving other massage therapies; having other developmental; having acute infection diseases, hemorrhagic diseases, or dermatological problems; having any severe illness or medical condition that the investigator deems not appropriate to receive pediatric *tuina*. Exclusion criteria for parents are: having any severe psychiatric disorder (e.g. major depressive disorder); having difficulties to conduct massage therapy due to physical problems; having a score of Montreal Cognitive Assessment less than 22, indicating cognitive impairment. |
| Study type | Interventional |
|  | Allocation: randomized |
|  | Intervention model: parallel assignment |
|  | Masking: no blinding (subject, practitioner, investigator) |
|  | Primary purpose: effects |
|  | Pilot study |
| Date of first enrolment | N/A |
| Target sample size | 60 |
| Recruitment status | Not yet recruiting |
| Primary outcome(s) | Hyperactivity (Baseline, week 4, week 8) |
| Key secondary outcomes | Anxiety; sleep disturbance; parental stress (Baseline, week 4, week 8) |
