# Supplementary material for: Pediatric tuina for the treatment of attention deficit hyperactivity disorder (ADHD) symptoms in preschool children: study protocol for a pilot randomized controlled trial
Source: Pilot Feasibility Stud. 2020 Nov 5;6:169. doi: 10.1186/s40814-020-00704-z (PMC7643336; doi:10.1186/s40814-020-00704-z)
Supplement: Supplementary file 3 — Additional file 3: Sample of a pediatric tuina prescription. [file 40814_2020_704_MOESM3_ESM.docx]

**Additional file 3: Sample of a pediatric *tuina* prescription**

**Diagnosis**： Attention Deficit Hyperactivity Disorder

**TCM pattern**: Liver-kidney yin deficiency pattern

**Treatment (area, acupoint, manipulation, times)**:

1. Manipulations on the head and face：

- Tianmen acupoint, arc-pushing, 100 times
- Kangong acupoint, pushing, 100 times
- Taiyang acupoint, kneading, 100 times
- Erhougaogu acupoint, kneading, 100 times

1. Manipulations on the upper limbs and lower limbs：
   - Shouyinyang acupoints, arc-pushing, 50 times
   - Ganjing acupoint, pushing, 300 times
   - Xiaochang acupoint, pushing, 300 times
   - Sanyinjiao acupoint, kneading, 30 times
2. Manipulations on the chest and abdomen：

- Tianshu acupoint, kneading, 50 times

**Reminder**:

- The treatment should be delivered at least every other days;
- Medium (such as water, sesame oil) have to be used during intervention delivery in order to protect the skin of your child.
