# Supplementary material for: Pediatric tuina for the treatment of attention deficit hyperactivity disorder (ADHD) symptoms in preschool children: study protocol for a pilot randomized controlled trial
Source: Pilot Feasibility Stud. 2020 Nov 5;6:169. doi: 10.1186/s40814-020-00704-z (PMC7643336; doi:10.1186/s40814-020-00704-z)
Supplement: Supplementary file 4 — Additional file 4: Parent logbook of pediatric tuina group. [file 40814_2020_704_MOESM4_ESM.docx]

**Additional file 4: Parent logbook of pediatric *tuina* group**

Date：**_______________________**

| **Code** | **Part of bady** | **acupoint** | **manipulation** | **Frequency** | **Times** | **Finish or not** | |
| --- | --- | --- | --- | --- | --- | --- | --- |
| 1 | Head and face | **Tianmen** | Arc-pushing | 200-300times/min | 100 times | 🞏 Yes | 🞏 No |
| 2 |  | **Kangong** | Pushing | 200-300 times/min | 100 times | 🞏 Yes | 🞏 No |
| 3 |  | **Taiyang** | Kneading | 80-120 times/min | 100 times | 🞏 Yes | 🞏 No |
| 4 |  | **Erhougaogu** | Kneading | 120-150 times/min | 200 times | 🞏 Yes | 🞏 No |
| 5 | Upper limbs and lower limbs | **Shouyinyang** | Arc-pushing | 80-120 times/min | 50 times | 🞏 Yes | 🞏 No |
| 6 |  | **Ganjing** | Pushing | 200-300 times/min | 300 times | 🞏 Yes | 🞏 No |
| 7 |  | **Xiaochang** | Pushing | 200-300times/min | 300 times | 🞏 Yes | 🞏 No |
|  |  | **Sanyinjiao** | Kneading | 120-150times/min | 30 times | 🞏 Yes | 🞏 No |
| 8 | Chest and abdomen | **Tianshu** | Kneading | 120-150times/min | 50 times | 🞏 Yes | 🞏 No |

**Reminder: Intervention should be delivered for at least every other day.**
