# Supplementary material for: Pediatric tuina for the treatment of attention deficit hyperactivity disorder (ADHD) symptoms in preschool children: study protocol for a pilot randomized controlled trial
Source: Pilot Feasibility Stud. 2020 Nov 5;6:169. doi: 10.1186/s40814-020-00704-z (PMC7643336; doi:10.1186/s40814-020-00704-z)
Supplement: Supplementary file 5 — Additional file 5: Parent logbook of parent-children interaction group. [file 40814_2020_704_MOESM5_ESM.docx]

**Additional file 5: Parent logbook of parent-children interaction group**

Date：**_______________________**

| **Code** | **Part of body** | **Finish or not** | |
| --- | --- | --- | --- |
| 1 | The relaxation of the forearm muscles | 🞏 Yes | 🞏 No |
| 2 | Relaxation of upper arm muscles | 🞏 Yes | 🞏 No |
| 3 | Relax the shoulder muscles | 🞏 Yes | 🞏 No |
| 4 | Relaxation of neck muscles（Part 1） | 🞏 Yes | 🞏 No |
| 5 | Relaxation of neck muscles（Part 2） | 🞏 Yes | 🞏 No |
| 6 | Relaxation of the back muscles | 🞏 Yes | 🞏 No |
| 7 | Relaxation of leg muscles（Part 1） | 🞏 Yes | 🞏 No |
| 8 | Relaxation of leg muscles（Part 1） | 🞏 Yes | 🞏 No |

**Reminder：Please repeat each set of exercise for at least two times every other day.**
